# A Supramolecular Trap to Increase the Antibacterial Activity of Colistin

**DOI:** 10.1002/anie.201912137

**Published:** 2020-01-07

**Authors:** Fang‐Hsuean Liao, Te‐Haw Wu, Chun‐Nien Yao, Shu‐Chen Kuo, Chun‐Jen Su, U‐Ser Jeng, Shu‐Yi Lin

**Affiliations:** ^1^ Institute of Biomedical Engineering and Nanomedicine National Health Research Institutes (NHRI) Zhunan Town Miaoli County 35053 Taiwan; ^2^ National Institute of Infectious Diseases and Vaccinology, NHRI Zhunan Town Miaoli County 35053 Taiwan; ^3^ National Synchrotron Radiation Research Center Hsinchu 30076 Taiwan

**Keywords:** antibiotics, endotoxin, Gram-negative bacteria, gold nanosheets, supramolecular chemistry

## Abstract

A strong interaction between colistin, a last‐resort antibiotic of the polymyxin family, and free lipopolysaccharide (LPS, also referred to as endotoxin), released from the Gram‐negative bacterial (GNB) outer membrane (OM), has been identified that can decrease the antibacterial efficacy of colistin, potentially increasing the dose of this antibiotic required for treatment. The competition between LPS in the GNB OM and free LPS for the interaction with colistin was prevented by using a supramolecular trap to capture free LPS. The supramolecular trap, fabricated from a subnanometer gold nanosheet with methyl motifs (SAuM), blocks lipid A, preventing the interaction between lipid A and colistin. This can minimize endotoxemia and maximize the antibacterial efficacy of colistin, enabling colistin to be used at lower doses. Thus, the potential crisis of colistin resistance could be avoided.

## Graphical abstract

**Caught in a trap**: The antibiotic colistin targets lipopolysaccharide (LPS) in the Gram‐negative bacterial (GNB) membrane. This interaction is disrupted by free LPS released during infection (path a). A methylated gold nanosheet (SAuM) binds to free LPS, preventing free LPS from binding colistin and reducing endotoxemia (path b). This increases the antibacterial efficacy of colistin, decreasing both the required dose and the risk of resistance.

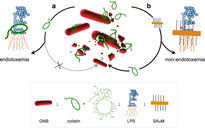

Antibiotic resistance has become an emerging challenge to global public health that should encourage people in general and health care providers in particular to realize the severe dangers stemming from antibiotic misuse.^1^, ^2^ Under antibiotic pressure, susceptible subpopulations of bacteria can spontaneously manipulate their own survival mechanisms by causing transient genomic instability to avoid death initiation, which can progressively move a given bacterial community toward an irreversible mutation event.^3^ In particular, the genomic mutation of Gram‐negative bacteria (GNB) subjected to a high dose of polymyxin E, also known as colistin, could result in a change in the structure of the lipopolysaccharide (LPS) on the outer leaflet of the outer membrane of GNB, potentially leading to antibiotic resistance.^4^ Colistin, a last‐resort antibiotic against GNB,^5^ strongly interacts with LPS and then rapidly penetrates the inner membrane of the GNB for efficient bacterial killing;^6^ therefore, changes in the LPS structure could decrease the antibacterial efficacy of colistin. There are no immediate threats of bacterial resistance to colistin, most likely due to the unique nephrotoxicity and limited therapeutic window of colistin that currently make it unfavorable for clinical use.^5^ Findings have suggested that, in contrast to other antibiotics, colistin might neutralize LPS.^7^ LPS is released upon GNB death and is known to cause endotoxemia.^8^, ^9^ Recently, various modification and formulation strategies have been attempted to resolve the toxicity issue limiting the use of colistin,^10^ and if any of these strategies ultimately work, it is expected that the therapeutic spectrum of colistin and the frequency with which it is used will be considerably expanded. Thus, in the future, humans could gradually come to depend on colistin to combat the bacteremia and endotoxemia caused by GNB. In that context, the potential crisis of colistin resistance could become a reality. In the present study, we demonstrate that a supramolecular trap fabricated from a subnanometer gold nanosheet with methyl motifs (SAuM) could effectively bind to the LPS released from GNB in order to not only avoid endotoxemia but also to prevent the interaction between colistin and free LPS, thereby boosting the low‐dose antibacterial activity of colistin.

Figure 1 shows that colistin may achieve low‐dose antibacterial activity against GNB by escaping from being hijacked by free LPS. The supramolecular interaction between colistin and free LPS may be stronger than that between colistin and GNB (Figure 1, path a). Thus, the competition between free LPS and GNB for the interaction with colistin could reduce the antibacterial effects of colistin. Colistin has been found to associate with LPS,^11^ therefore precisely targeting the active site of LPS, namely lipid A, could prevent endotoxemia. In other words, if it is possible to find an LPS neutralizer to intervene in the competition between bacteria and free LPS for colistin, the effectiveness of colistin treatment would be improved and the risk of endotoxemia can be reduced. It was recently found that SAuM is able to precisely target free LPS by binding to and compressing the packing density of lipid A, thereby significantly reducing the risk of endotoxin‐induced sepsis.^12^ Thus, we speculated that SAuM may also act as an efficient supramolecular trap to directly capture free LPS, which could increase the steric hindrance of LPS, thereby preventing its interaction with colistin (Figure 1, path b). This could allow the therapeutic window of colistin to be expanded to low‐dose concentrations for the treatment of GNB infection, while also minimizing the risk of endotoxemia.

**Figure 1:**
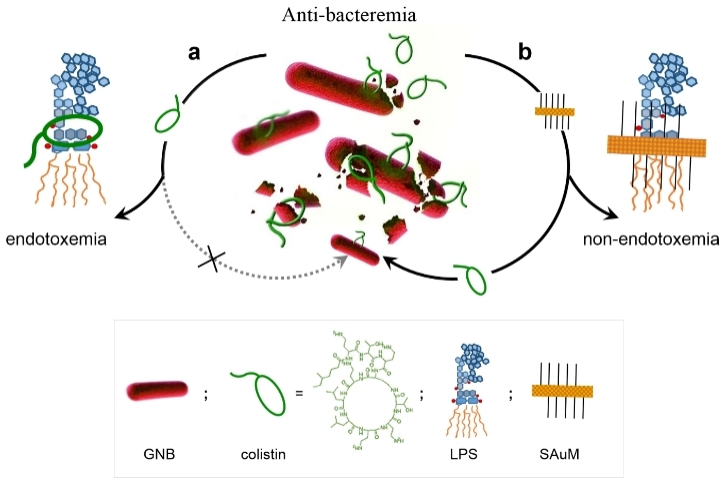
A simple illustration describing how the supramolecular trap restores the anti‐bacterial activity of colistin. Free LPS can block the intended function of colistin (path a) but can be sequestered by SAuM in the circulating blood (path b), thus boosting the killing efficiency of the antibiotic against GNB while also minimizing endotoxemia.

To test if the presence of LPS affects the antibacterial efficiency of colistin, a competition test between LPS and bacteria was conducted. As expected, Figure 2 a shows that colistin (at doses of 1.0 μg mL^−1^ and 2.0 μg mL^−1^) failed to effectively kill *Escherichia coli* (a GNB) in the presence of even a low concentration of only 0.1 nm LPS. Surprisingly, however, the antibacterial activity of colistin (at doses of 1.0 μg mL^−1^ and 2.0 μg mL^−1^) was restored in the absence of LPS (Figure 2 a, blank bar). These results strongly indicate that free LPS and bacteria compete for binding with colistin. Further direct evidence showed that the critical micelle concentration (CMC) values of LPS significantly increased from 4 μg mL^−1^ to 15 μg mL^−1^ in the presence of colistin (Supporting Information, Figure S1). These results suggest that colistin can associate with LPS to suppress LPS self‐assembly. Notably, LPS is prone to self‐assembly, forming various aggregates that are directly implicated in its bioactivity.^13^ Otherwise, in the presence of other antibiotics, including penicillin, ceftazidime, and rifampicin, the CMC values of LPS are approximately 3–5 μg mL^−1^ and are similar to LPS‐alone (4 μg mL^−1^; Supporting Information, Figure S1). This clearly illustrates that only colistin strongly interacts with LPS, while the other antibiotics tested do not. Thus, it is essential to further study how it might be possible to intervene in the competition between free LPS and bacteria for interacting with colistin. Recently, some supramolecules have been reported to directly target antibiotics and thereby switch‐on/off their activity,^14^, ^15^ but there have still been no findings indicating that the antagonism of free LPS restores colistin activity. Due to the lack of an LPS antagonist,^16^ we were inspired by the finding that SAuM increases the CMC value of LPS to 17 μg mL^−1^ (Supporting Information, Figure S1) and therefore may act as a supramolecular trap to capture free LPS, thereby disrupting the interaction between free LPS and colistin. The standard test of minimal inhibitory concentration (MIC) for antimicrobial susceptibility was conducted.^17^ We found that the MIC of colistin in the presence of SAuM is 0.5 μg mL^−1^ (Figure 2 b,c), that is, the activity of a low dose of colistin,^18^ is restored in the presence of SAuM. Confocal and scanning electron microscopy (SEM) images (Supporting Information, Figure S2) also support this observation. Furthermore, the MIC of colistin in the presence of SAuM is the same for colistin‐resistant and colistin‐nonresistant GNB (Supporting Information, Figure S3). The results strongly suggest the SAuM could indeed act as a trap to help colistin escape from being hijacked by LPS, thus boosting its low‐dose antibacterial efficiency and providing a means to avoid the risk of colistin resistance.

**Figure 2:**
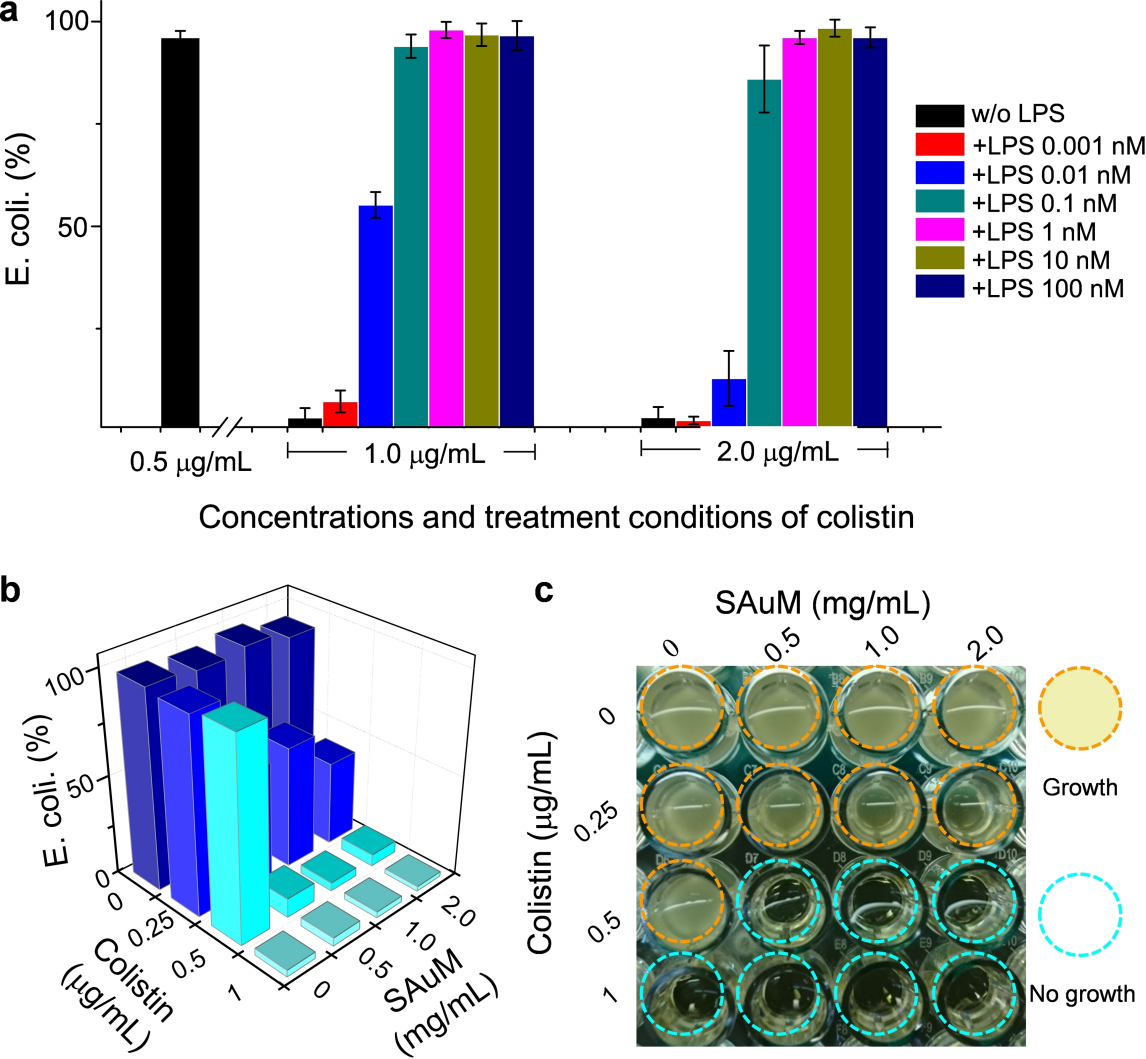
Assessment of the *E. coli* killing efficiency of colistin. a) The presence of LPS can dramatically decrease the level of *E. coli* killed by colistin at concentrations of 1.0 and 2.0 μg mL^−1^. b,c) The addition of SAuM to neutralize free LPS restores the antibacterial activity of colistin.

The binding specificity assay results showed that the interaction between LPS and SAuM is actually stronger than that between LPS and colistin (Figure 3 a, line iii versus line iv), which shows that SAuM is a strong competitor of colistin for binding to free LPS. The driving force of SAuM to bind with LPS could be deduced from its special geometry and the adhesion of its decoration motifs, structural details of which have been published previously.^12^ In brief, SAuM consists mostly of Au_8_‐dominated nanoclusters (the nanoclusters mainly consisting of eight gold atoms) with typical excitation and emission peaks at approximately 390 nm and approximately 460 nm,^19^ respectively (Figure 3 b). The core dimensions of SAuM are less than 1 nm, allowing it to potentially have a sheet‐like geometry,^12^, ^20^, ^21^ which in turn makes it prone to forming microcrystalline structures via layer‐by‐layer stacks on copper grids (Supporting Information, Figure S4) and showing well‐ordered arrangement (Figure 3 c) in terms of the neighbor distance (approximately 0.288 nm)^22^ of the gold atoms (inset of Figure 3 c). Unlike other nanoclusters, which have curvatures with various orientations, the sheet‐like structure of SAuM can precisely bind the lipid A of LPS to form a steric wall that efficiently inhibits colistin binding. Direct evidence of this can be observed by the short‐range ordered packing of lipid A with characteristic LPS d‐spacing offsets (estimated from the hump center; Figure 3 d), which originates from the hydrocarbon chain–chain distance in individual lipid A molecules (the active site of LPS, inset model of Figure 3 e), which is also known to be relevant to the biological activity of LPS.^13^, ^23^ Figure 3 e summarizes that the d‐spacing changed from approximately 4.19 Å to approximately 3.88 Å in the presence of SAuM. This small yet observable difference indicates a tighter packing of lipid A of LPS, giving the LPS a weakly bioactive conformation. In contrast, the d‐spacing value of LPS in the presence of colistin (approximately 4.17 Å) was almost identical to that of LPS in the absence of colistin (approximately 4.19 Å). It is therefore not expected that LPS could also bind to colistin (Figure 3 a, line i) to the degree that it does to SAuM (Figure 3 a, line ii). There are no significant d‐spacing changes when LPS binds with colistin, strongly indicating that the interaction between colistin and LPS could not favor the site of lipid A.^11^ Interestingly, when LPS was in the presence of both colistin and SAuM, only the SAuM could bind with LPS (Figure 3 a, line iii versus line iv), and the d‐spacing value of lipid A in the presence of SAuM (approximately 3.87 Å) was also smaller that in the absence of SAuM (approximately 4.17 Å). It is noteworthy that the presence of both SAuM and ceftazidime, an antibiotic that induces LPS release,^24^ also results in a similar trend in d‐spacing value (approximately 3.98 Å) that might correlate to a significant decrease in the MIC of ceftazidime (Supporting Information, Figure S5). These results indicate that SAuM could sufficiently target lipid A of LPS, which could increase the steric hindrance of LPS and thus directly block the attraction between antibiotics and LPS.

**Figure 3:**
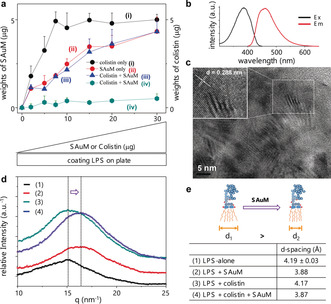
Confirmation of the competition between colistin and SAuM for binding to free LPS. a) The binding assay shows that once SAuM was present in the mixture, colistin could no longer associate with LPS. The association test was carried out in a dose‐dependent manner (lines i and iv follow the right *y*‐axis, and lines ii and iii follow the left *y*‐axis). b) Photoluminescence spectra of SAuM. c) TEM image showing the core geometry of SAuM. d) The packing density of a single lipid A was higher in the presence of SAuM than in the absence of SAuM as shown by the decrease in d‐spacing, regardless of whether colistin was present. q is the scattering factor. The dotted line and arrow show the change in d‐spacing. The LPS model presented in (e) shows the d‐spacing changes from d_1_ to d_2_, indicating that the tightening of the lipid chain packing of lipid A changed from loose to tight. The table within (e) shows the d‐spacing of lipid A under each condition, in which uncertainty value obtained from q‐peak fitting process is 0.03. Since the changes are very tiny, the uncertainty range can provide the confidence on the differences in d‐values observed.

Next, it is essential to check the antagonistic activity of SAuM in LPS‐challenged mice. After an LPS challenge, mice rapidly die, typically within one day. However, the survival rate of LPS‐challenged mice increased to 90 % with SAuM treatment (Supporting Information, Figure S6) and this survival rate is only slightly lower than that of SAuM‐treated mice that were not challenged with LPS (100 %). The results illustrated that SAuM is an excellent LPS antagonist and also possesses excellent biocompatibility. The hydrodynamic size of SAuM of around 2 nm^12^ is smaller than 5.5 nm, which is the maximum particle size that can be efficiently excreted by the urinary system,^25^ meaning that SAuM can be efficiently cleared by the renal system. Thereafter, we used GNB‐infected mice to investigate the aforementioned speculation, which is, whether SAuM can help colistin to escape from LPS hijack and, consequently, increase the antibacterial efficacy of colistin in the GNB‐infected mice. Notably, SAuM has a long half‐life in blood (15 h),^12^ similar to that of colistin (13 h),^26^ and did not possess anti‐GNB activity, unlike other nanoparticles that could cause drug resistance of new type.^27^, ^28^ As expected, Figure 4 shows that the survival rate of GNB‐infected mice injected with colistin alone was approximately 50 % after 5 days. In comparison, the percentage survival of GNB‐infected mice on day 5 post‐treatment with SAuM and colistin increased to approximately 60 % and approximately 90 %, depending on the SAuM dose. This significant improvement was due to the role of SAuM in interfering with the interaction between colistin and free LPS. Taken together, the competition for colistin between free LPS and GNB was identified and successfully addressed by the supramolecular trap, SAuM, to restore the antibacterial efficacy of colistin.

**Figure 4:**
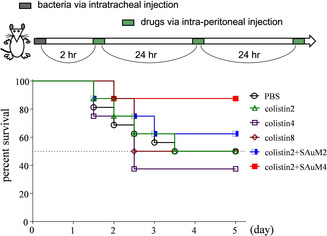
Assessment of the anti‐bacteremia effect of colistin combined with SAuM. Two hours after the intratracheal injection of bacteria, infected mice were injected intraperitoneally three times (at 3‐day intervals) with various dosages of colistin or the mixture of colistin and SAuM. The injection dosages of colistin were 2, 4, and 8 mg kg^−1^/injection, referred to as colistin2, colistin4, and colistin8, respectively. The injection dosages of SAuM were 2 and 4 mg kg^−1^/injection, referred to as SAuM2 and SAuM4, respectively. The bacteria injected were *Acinetobacter baumannii* ATTC17 978 (5×10^8^ CFU per mouse). The combination of colistin and SAuM can more efficiently protect the mice with bacterial infection from bacteremia than colistin alone. The dashed line on the Kaplan–Meier survival plot represents the survival of 50 % of the mice. The survival plot was analyzed by the Log‐rank test (*n*=8 each group; colistin2+SAuM4 vs. colistin, *P*=0.0585).

In summary, we can prevent the interaction between the antibiotic colistin and free LPS by using a supramolecular trap fabricated from subnanometer gold nanosheets. The capture and deactivation of free LPS not only minimizes the degree of endotoxemia but also boosts the low‐dose antibacterial activity of colistin which, in turn, reduced the degree of death due to GNB bacteremia. The LPS‐antagonistic activity of this supramolecular trap provides an effective steric hindrance by sealing off lipid A of free LPS to prevent the interaction between colistin and free LPS. Without the interference of free LPS, colistin can maintain its maximum antibacterial activity at a low dose, minimizing both endotoxemia and bacteremia. The supramolecular trap thus offers a potential strategy for avoiding the risk of colistin resistance.

## Conflict of interest

The authors declare no conflict of interest.

## Supporting information

As a service to our authors and readers, this journal provides supporting information supplied by the authors. Such materials are peer reviewed and may be re‐organized for online delivery, but are not copy‐edited or typeset. Technical support issues arising from supporting information (other than missing files) should be addressed to the authors.

Supplementary
