# Supplementary material for: A Supramolecular Trap to Increase the Antibacterial Activity of Colistin
Source: Angew Chem Int Ed Engl. 2020 Jan 7;59(4):1430–4. doi: 10.1002/anie.201912137 (PMC7687082; doi:10.1002/anie.201912137)
Supplement: Supplementary file 1 — Supplementary [file ANIE-59-1430-s001.pdf]

Angewandte Chemie
Eine Zeitschrift der Gesellschaft Deutscher Chemiker

Supporting Information

A Supramolecular Trap to Increase the Antibacterial Activity of Colistin

*Fang-Hsuean Liao+, Te-Haw Wu+, Chun-Nien Yao+, Shu-Chen Kuo, Chun-Jen Su, U-Ser Jeng, and Shu-Yi Lin**

anie_201912137_sm_miscellaneous_information.pdf

Materials and methods

Figure S1-S6

Supplementary references

Materials and Methods

Materials. The G_4NH_2 dendrimer, G_4OH dendrimer, $HAuCl_4$, and LPS (*Escherichia coli* 0111:B4) were purchased from Sigma, Inc. (San Diego, CA, USA); an MWCO membrane filter was purchased from Millipore (PES membrane); WST-1 was obtained from Dojindo Laboratories (Kumamoto, Japan); and an anion exchange resin was purchased from (Merck, Fractogel® EMD TMAE Hicap).

Synthesis of SAuM. The subnanometer nano-gold (SAuM) were synthesized according to a previously published method.[1] First, varying amounts of $HAuCl_4$ (Sigma-Aldrich, 400 μL, 60 μmol, 150 mM) were added into 20 mL of deionized water containing the G_4NH_2 (Aldrich, 874 μL, 46 μmol, 10 wt % methanol solution). Each G_4NH_2 and $HAuCl_4$ mixed solution was then incubated at 4°C overnight before being irradiated with microwaves (CEM, Discover LabMate System, 300W/120°C for 30 min). After reduction, the precipitates and SAuM were filtered through a 3 KDa MWCO PES membrane filter (Millipore, Amicon Ultra), and the extra anion, such as $AuCl_4^-$, was removed by an anionic exchange chromatograph to obtain the purified SAuM precursor from G_4NH_2. Then, the internal tertiary amine groups and the surface amine groups of the dendrimer-encapsulated nano-gold (i.e. SAuM precursor, 63.6 mg, 4 μmol) were reacted with [2]methyl iodide (24 μl, 0.38 mmol) in dichloromethane/N,N'-dimethyl formamide/H_2O (4 mL/5 mL/1 mL) at room temperature or 37°C overnight, respectively. Each reaction mixture was extracted by dichloromethane 3 times and then lyophilized to derive two yellow gel-like compounds. Notably, the decoration numbers of methyl group were 96. As validation of this decoration, the ^{1}H NMR spectra showed a main peak at 2.5~4 ppm from the methyl groups when compared to the precursor of SAuM. All samples were dissolved in D_2O as a solvent for measurement.

The measurement of size and hydrodynamic diameter of SAuM. Each sample was mounted on a carbon film. The grid was dried prior to transmission electron microscopy measurements (JEOL JEM-3000F, Japan) at 300 kV. As reported in our previous work,[3] the transmission electron microscope (TEM) images showed that SAuM favors a layer-by-layer sticking in copper grid. Since the peak of emission wavelength at 460 confirms an Au_8-dominating nanocluster as a main product, the core size of a single SAuM is less 1 nm that cannot be observed by High-resolution TEM (HRTEM) resolution. If used dynamic light scattering (DLS) can provide an inaccuracy values in diameter measurement, the particle size distribution showing in 0.6~1.1 nm is very close the scattering limitation. Thus, we determined to use polyacrylamide gel electrophoresis (PAGE) to validate the hydrodynamic size of SAuM around 2 nm, which was published in our previous work for the development of synthetic strategy.[4]

Small-angle X-ray scattering (SAXS) and grazing-incidence wide-angle X-ray scattering (GIWAXS) measurements. GIWAXS data for the LPS were acquired at the 23A SWAXS beamline of the Taiwan Light Source of the National Synchrotron Radiation Research Center (NSRRC) in Taiwan.[5] Thin-film samples for the GIWAXS measurements were prepared by drop casting on a silicon wafer. With a 15 keV (wavelength λ = 0.8267 Å) beam, a sample-to-detector distance of 132 mm, and an incident angle of 0.2^{o}, GIWAXS data were collected using a CMOS flat panel X-ray detector C9728DK (52.8 mm square). The d-spacing (= $2\pi/q_c$) for lipid A was deduced from the WAXS peak position q_c observed. The SAXS data, collected using a Pilatus 1M-F detector, for the sample solutions were used to extract the zero-angle intensity I_o (q = 0) and radius of gyration R_g of the LPS micelles, on the basis of Guinier approximation[6] as that shown in Fig. S1a. Critical micellar concentration (CMC) was then extracted from the intercept of the

linear regression fitting of the concentration-dependent zero-angle intensity I_o ($q = 0$) as that shown in Fig. S1a.

A binding assay for the detection of LPS and SAuM. 96-well plates (Costar 3991, Corning Inc.) were coated with the fixed concentration of LPS 30 ug/ml in 0.1M Na_2CO_3 buffer containing 0.02M EDTA and heated at 37°C for 200 min. After that, the coating plates were washed with deionized water and dried for 16 hr. Then the plates were blocked with 1% BSA in PBS at 37°C for 30 min and washed with 0.1% BSA in PBS. To examine the binding of colistin and SAuM to LPS, we added fluorophore-conjugated colistin and SAuM concentrations to the LPS-coated plates. Then the plates were washed with 0.1% BSA in PBS three times. Finally, 100 µl of deionized water was added in each coated well and an ELISA reader (SpectraMax M2, Moleculardevices Inc.). Then the calibration curve was used to determine the weight of the combination with the LPS on the plate. SAuM and Alexa Fluor 647 were measured at Ex/Em 390nm/460nm and Ex/Em 630nm/670nm, respectively. Fluorophore-conjugated colistin was prepared by incubating colistin with Alexa Fluor 647 NHS ester (Sigma-Aldrich) at a 5 to 1 molar ratio in water for 72 hr.

Anti-bacterial test. Three kinds of gram-negative bacteria (denoted GNB), including Escherichia coli (denoted as DH5α), colistin-resistant Klebsiella pneumonia (denoted as Colistin R kpn) and Acinetobacter baumannii (denoted as AB) were used in vitro and in vitro level, respectively. The E. coli were grown in 1 ml of LB Broth (BD Inc.) in a 14 ml culture tube (FALCON Inc. 352001). In the process of bacteria growth, first different concentrations of LPS, colistin (Sigma-Aldrich), and SAuM were added into the LB solution, and then 40ul of E coli was added. Each mixture was

shaken for 16 hours at 37°C during the incubation, then 100 μl of the culture was placed into 96-well plates (REF 9018, Corning Inc.) Afterward, these samples were measured using an ELISA reader (SpectraMax M2, Molecular devices Inc.) to collect the absorbance at 600 nm. Alternatively, In-vitro microbiological tests were used to determine the minimal inhibition concentration (MIC) of antibiotics in the absence and presence of SAuM. We have check two categories, including (1) antibiotic-non-induced LPS release and (2) antibiotic-induced LPS release. We first test cefpodoxime and rifampicin, belong to the category without LPS release, that can only inhibit RNA polymerase and cell wall synthesis, respectively, for anti-infection. As expected, no any improved in the killing efficacy in the presence of SAuM. On the contrary, antibiotic (colistin and ceftazidime) of induced LPS release can be reduced their MIC in the assistance of SAuM. Ceftazidime treatment has be found to release higher abundant LPS compared to other beta-lactam antibiotic.[7] Despite the CMC of ceftazidime in the presence of LPS has no significant increase, the packing density (d-spacing) of a single lipid A in the co-existence of SAuM and ceftazidime could become denser that is similar to that of in the co-existence of SAuM and colistin. Meanwhile, the killing efficacy of ceftazidime in the assistance of SAuM was significantly augmented. The MIC of ceftazidime can be decreased to 100 ng/ml while helping by SAuM.

The measurement of bacterial images. Incubating Escherichia coli at 37°C for 16 hours, the bacterial density was adjusted to 0.6 OD (600 nm), then either colistin or SAuM or the mixture were added. The Escherichia coli will be incubating at 37°C again for 1 hours, than centrifuge the bacteria (25°C, 1000 rpm 10min), the precipitate was wash with water and centrifuging again. The precipitated bacteria were dried and observed using a scanning electron microscope (TM-1000,

HITACHI). Again, bacterial viability was performed LIVE/DEAD stain (Thermo Fisher) following the manufacturer's procedure and then imaged using the confocal microscope FV10i (Olympus).

Animals. Male 4-week-old C57BL/6JNarl mice were obtained from the National Laboratory Animal Center (Taipei, Taiwan). All mice were housed in a specific pathogen-free environment with moderate humidity and temperature at the Laboratory Animal Center of the National Health Research Institutes (NHRI, Miaoli, Taiwan), which is accredited by the Association for Assessment and Accreditation of Laboratory Animal Care International (AAALAC International). All animal experiments were approved by the NHRI's Institutional Animal Care and Use Committee.

In vivo endotoxemia or bacterima model. To examine the efficiency of colistin treatment in endotoxemic mice, we used the experimental LPS-induced sepsis model described in our previous study. Briefly, male C57BL/6JNarl mice received intraperitoneal (i.p.) injections with a lethal dose of 25 mg/kg LPS (Escherichia coli O111:B4, InvivoGen) in 100 μL PBS from a 29 gauge needle. In order to avoid other contamination of bacterial debris, ultrapure LPS can used in anti-endotoxemia test again. In the SAuM treatment groups, the mice were injected with SAuM (75 mg/kg body weight) twice before and after the LPS injection at 30-minute intervals, while mice that received two injections of SAuM with a 60-minute interval served as the control group. To investigate the interaction of colistin and SAuM, 6-week-old mice were infected with acinetobacter *baumannii* ATCC 17978 (5×10^8 CFU/mouse) placed into lungs through an intra-tracheal injection to induce pneumonia. At 2 hr post-infection, the first treatments of PBS, colistin (2 mg/kg/time)

and the mixture of colistin and SAuM were administered with intraperitoneal injections, once a day for 3 times. The mice were observed at 12-hr intervals for survival.

Statistical analyses. We used the GraphPad Prism program (v7.02) to conduct statistical analyses. The survival rate data were plotted using Kaplan-Meier curves and analyzed by the Log-rank (Mantel-Cox) test to calculate statistical differences.

Figure S1:

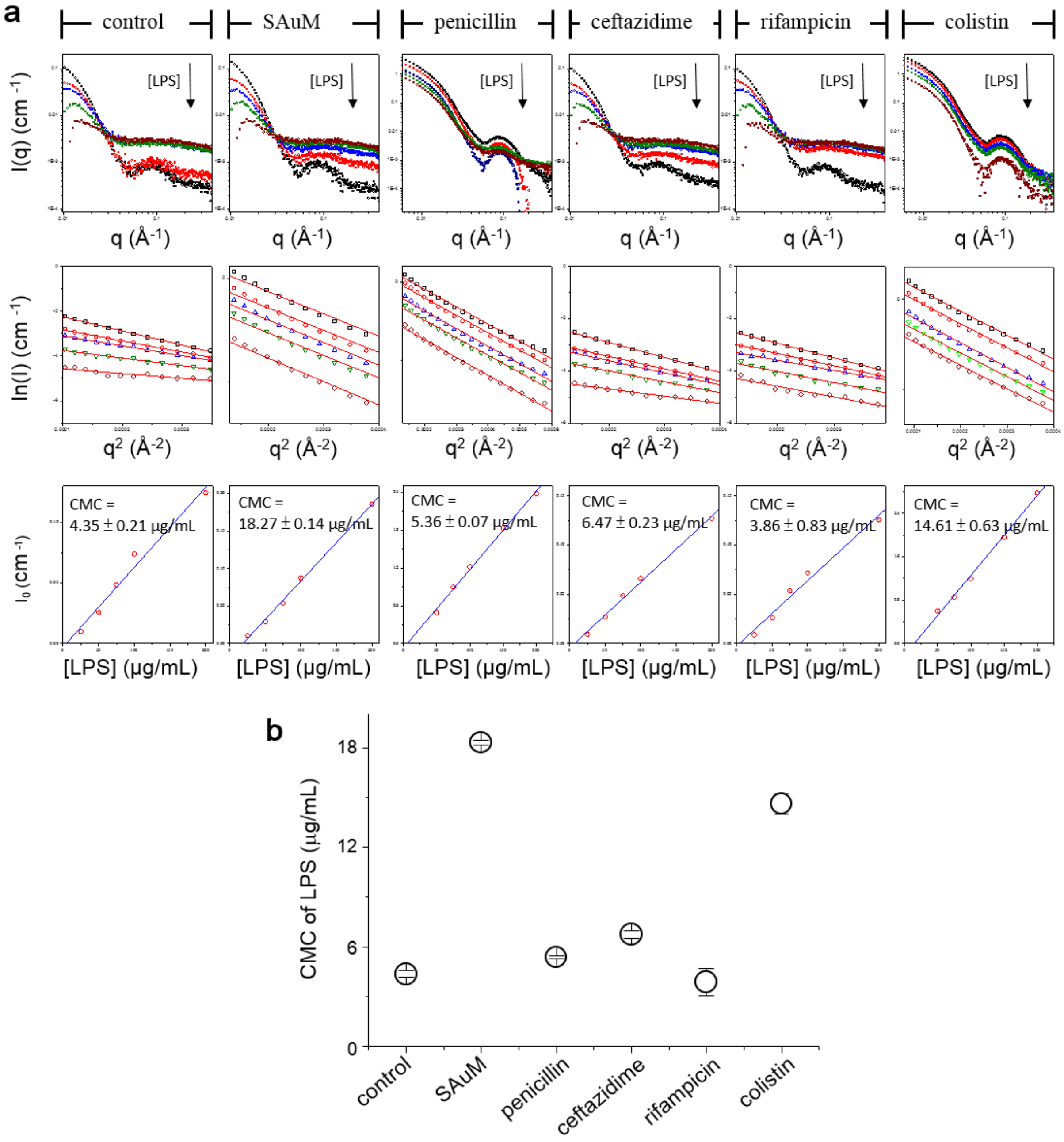

Figure S1 The measurement of CMC of LPS. (a) The plots of scattering intensities as a function of q, defined by $q = 4\pi\lambda^{-1} \sin(\theta)$ with the scattering angle 2θ and X-ray wavelength λ. These signals of nascent LPS aggregates show at different concentrations in presence of various antibiotics or SAuM. The CMC values of the third row of figure are with uncertainties (error bars).

(b) Summary of the changes in the CMC of LPS under different conditions as determined by SAXS measurements. The concentrations of LPS from top to bottom are 200 μg/mL, 100 μg/mL, 75 μg/mL, 50 μg/mL and 25 μg/mL.

Figure S2:

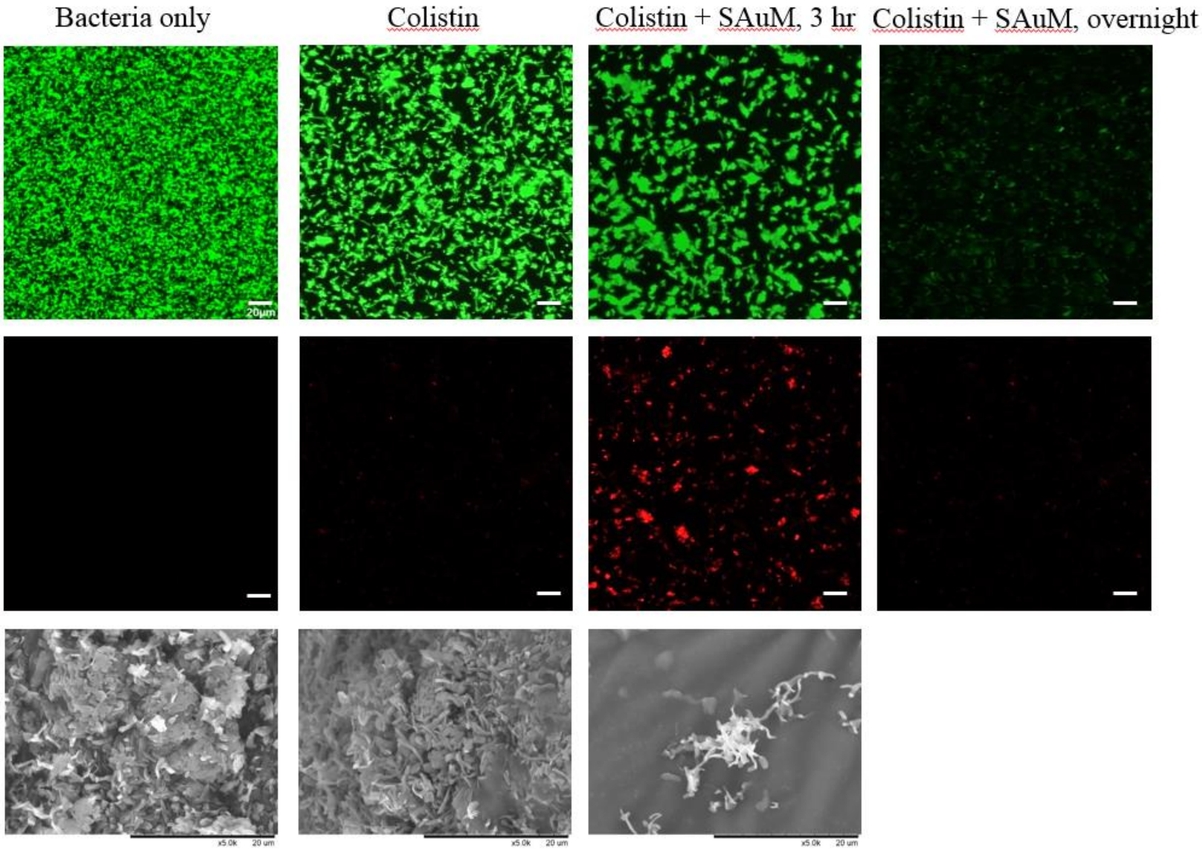

Figure S2 The difference of bacterial viability and morphology before and after treatment. Confocal images shows the green (SYTO9) and red (propidium iodide) staining for live/dead bacteria. Note that the SYTO9 (green) would stain the membranes of all bacteria regardless live and dead, but the propidium iodide (red) could only stain the membranes of dead bacteria. The treatment condition was same as the MIC test. SEM micrograph images displayed shrunken and deformed surface morphologywhile co-treated with colistin and SAuM, indicating cell death that was similar to a previous report.[8] Since increased the treatment time to overnight, no bacteria can be observed during SEM measurement.

Figure S3:

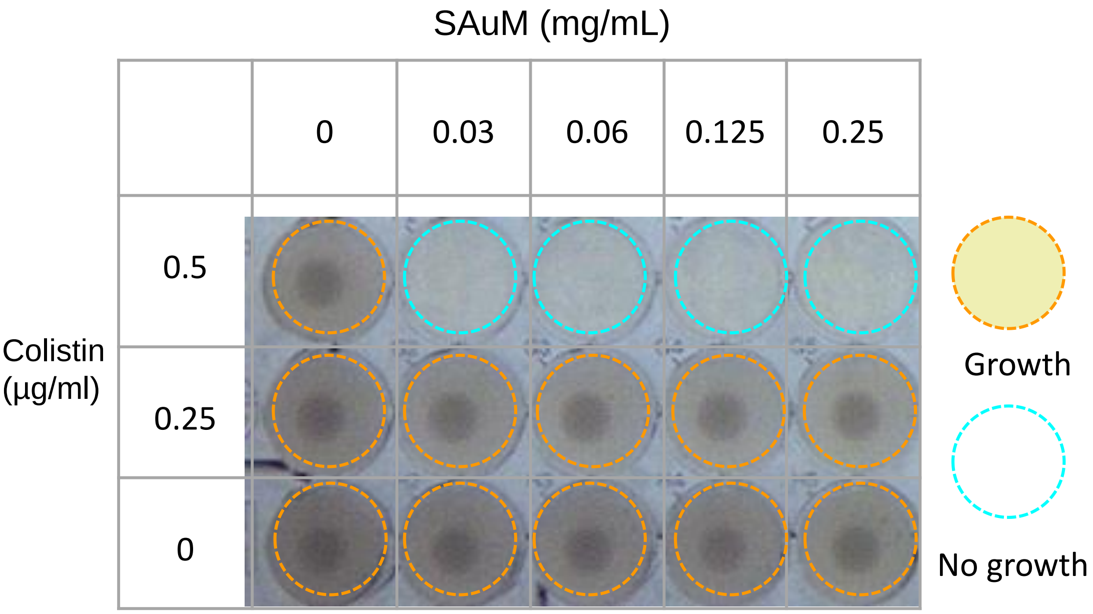

Figure S3 The killing efficiency of colistin-resistant GNB (colistin-resistant Klebsiella pneumonia). The first row shows that neutralized the interference of LPS by SAuM, the MIC of colistin in colistin-resistant Klebsiella pneumonia still appearred at 0.5 μg/mL as non-resistance GNB.

Figure S4:

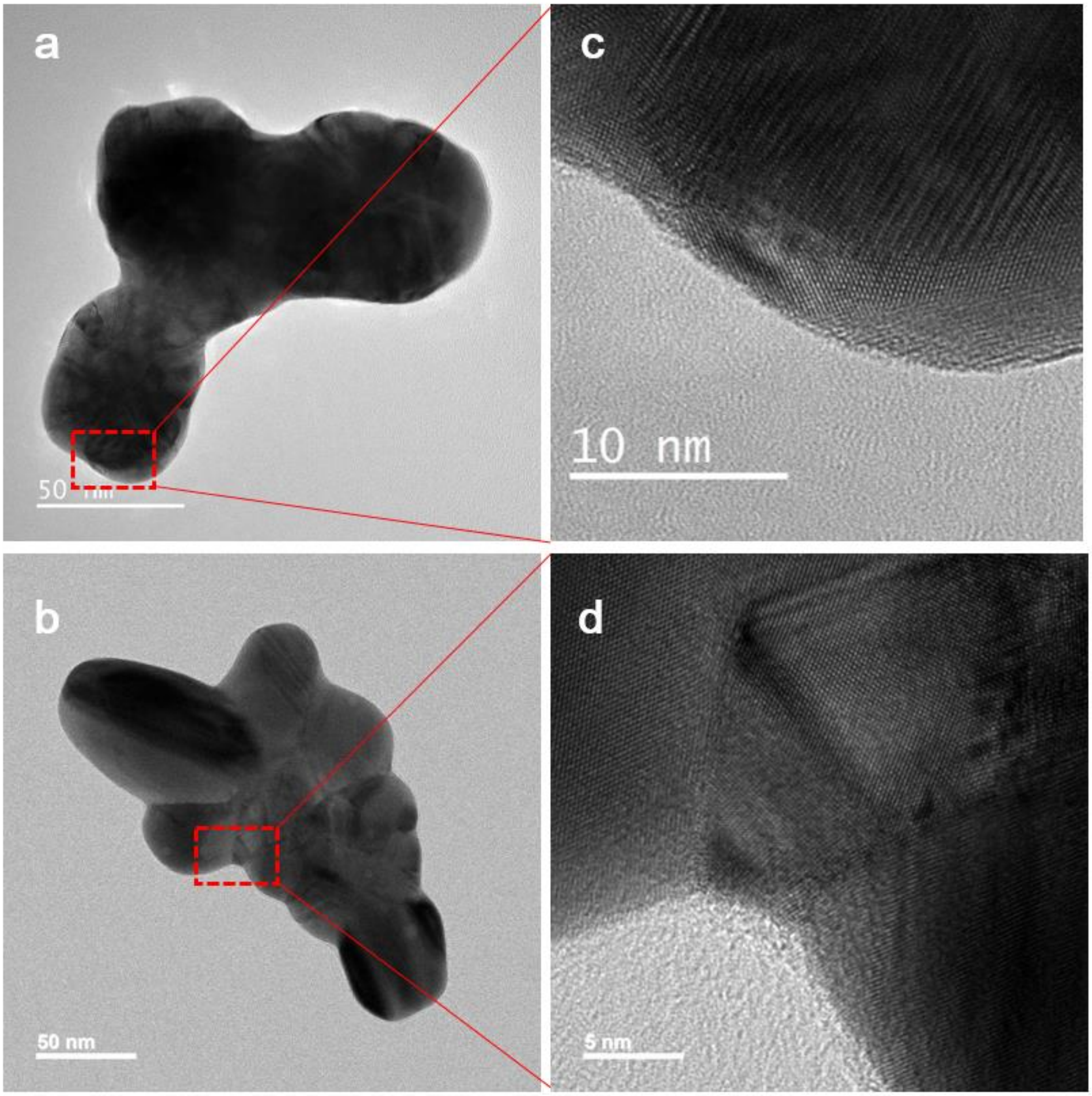

Figure S4 The microcrystalline structures of SAuM self-stacking. Two TEM images in (a) and (b) show that layer-by-layer stacks of SAuM can form microcrystalline structures of different shapes. (c) and (d) show clear atomic resolution of SAuM self-stacking, which was magnified from rectangles of (a) and (b) respectively. The gold atoms of SAuM can easily self-stack to form thin-films and a well-ordered alignment, the detailed aligment of SAuM self-stacking has been discussed and reported in our previous work.[3]

Figure S5:

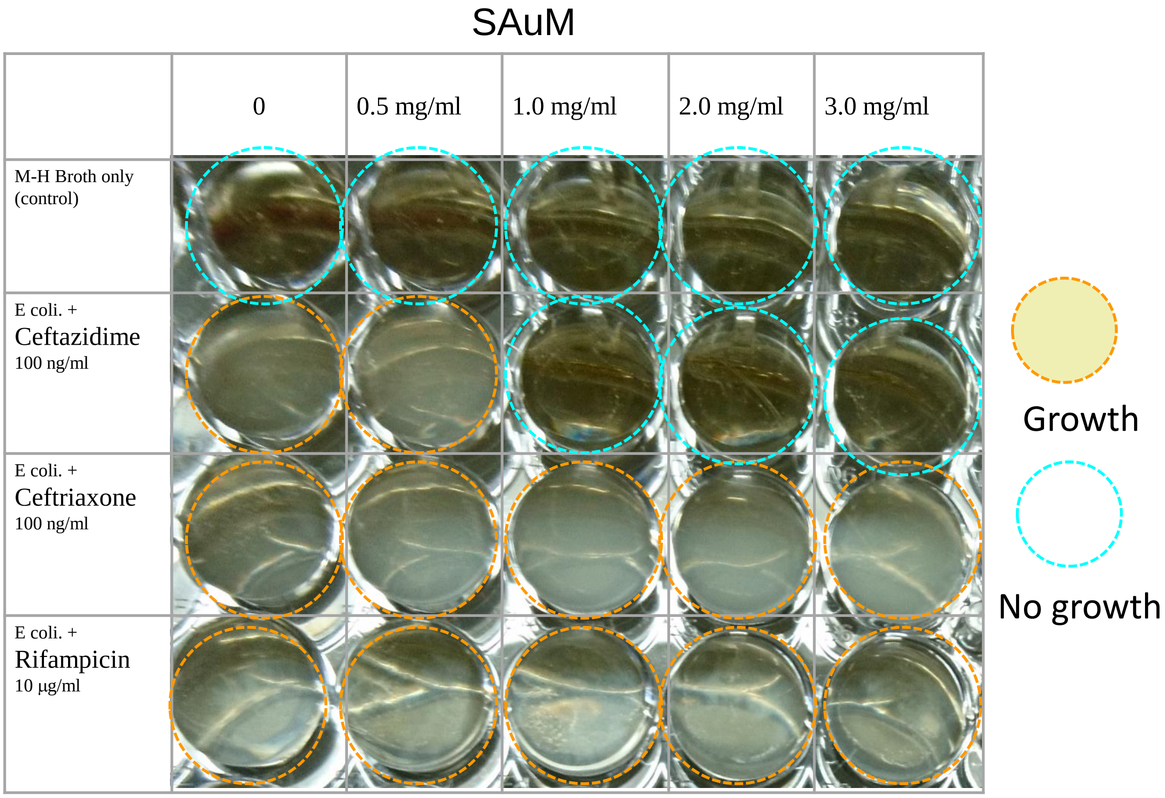

Figure S5 The killing efficiency of colistin-resistant GNB. The second row shows that the MIC of colistin the resistant GNB still appearred at 0.5 μg/mL while neutralized the interference of LPS by SAuM..

Figure S6

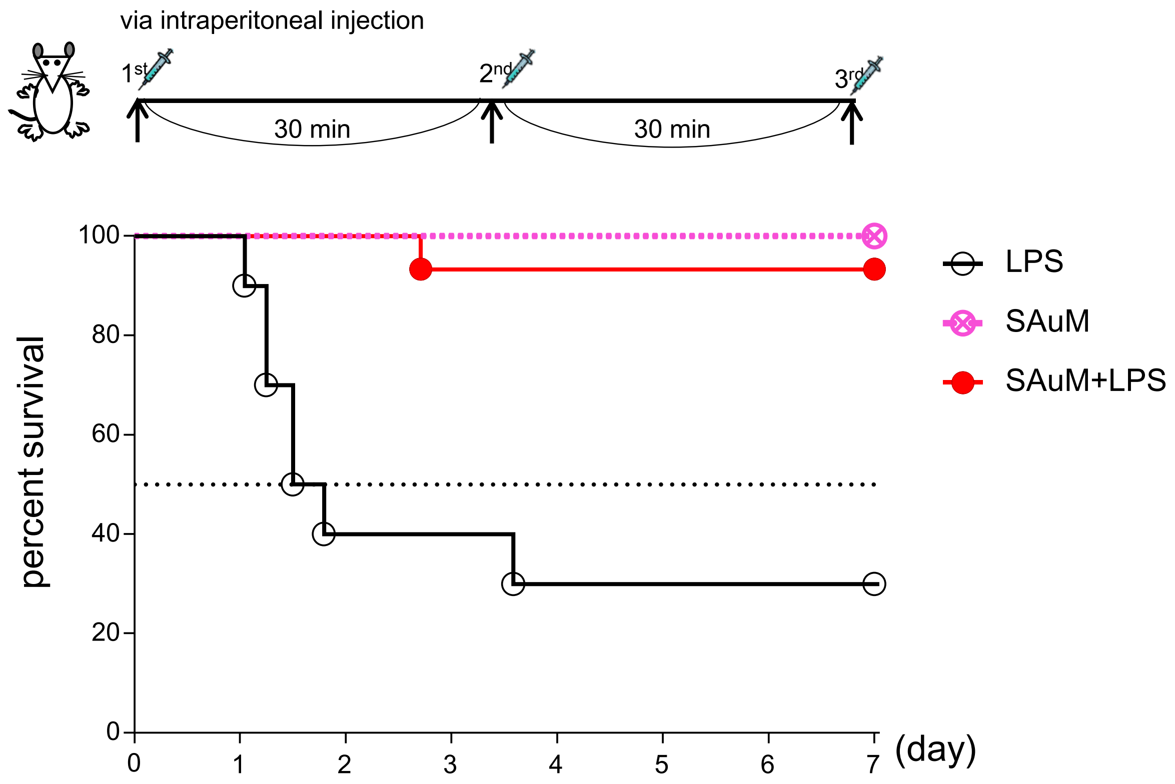

Figure S6 Assessment of the antagonistic activity of SAuM in LPS-challenge mice. The survival rates of LPS-challenged mice (25 mg/kg BW) subjected to the treatments with SAuM (75 mg/kg BW). The dash line represented the half percentage survival (LPS, n=10; SAuM, n=9; SAuM+LPS, n=15; SAuM+LPS vs. LPS: *P*=0.0004 (****P*<0.001); SAuM vs. LPS: *P*=0.002 (***P*<0.01)).

Supplementary References

[1] Y. C. Jao, M. K. Chen, S. Y. Lin, *Chem. Commun.* **2010**, *46*, 2626-2628.
[2] S. M. Wolff, J. V. Bennett, *N. Engl. J. Med.* **1974**, *291*, 733-734.
[3] F. H. Liao, T. H. Wu, Y. T. Huang, W. J. Lin, C. J. Su, U. S. Jeng, S. C. Kuo, S. Y. Lin, *Nano Lett.* **2018**, *18*, 2864-2869.
[4] C. T. Chien, C. Y. Liu, Z. W. Wu, P. J. Chen, C. L. Chu, S. Y. Lin, *J. Mater. Chem. B* **2014**, *2*, 6730-6737.
[5] U. S. Jeng, C. H. Su, C. J. Su, K. F. Liao, W. T. Chuang, Y. H. Lai, J. W. Chang, Y. J. Chen, Y. S. Huang, M. T. Lee, K. L. Yu, J. M. Lin, D. G. Liu, C. F. Chang, C. Y. Liu, C. H. Chang, K. S. Liang, *J. Appl. Crystallogr.* **2010**, *43*, 110-121.
[6] O. Glatter, O. Kratky, *Small angle x-ray scattering*, Academic Press, London, **1982**.
[7] J. J. Jackson, H. Kropp, *J. Infect. Dis.* **1992**, *165*, 1033-1041.
[8] B. Chandar, S. Poovitha, K. Ilango, R. MohanKumar, M. Parani, *Front Microbiol.* **2017**, *8*.
